# Bridging the Gaps in Atrial Fibrillation Management in the Emergency Department

**DOI:** 10.3390/jcdd12010020

**Published:** 2025-01-08

**Authors:** Brian Xiangzhi Wang

**Affiliations:** Department of Cardiology, Jersey General Hospital, Gloucester Street, St. Helier, Jersey JE1 3QS, UK; brian.wang15@imperial.ac.uk

**Keywords:** atrial fibrillation, emergency medicine, arrhythmia

## Abstract

Atrial fibrillation (AF) frequently presents in emergency departments (EDs), contributing significantly to adverse cardiovascular outcomes. Despite established guidelines, ED management of AF often varies, revealing important gaps in care. This review addresses specific challenges in AF management for patients in the ED, including the nuances of rate versus rhythm control, the timing of anticoagulation initiation, and patient disposition. The updated 2024 European Society of Cardiology (ESC) guidelines advocate early rhythm control for select patients while recommending rate control for others; however, uncertainties persist, particularly regarding these strategies’ long-term impact on outcomes. Stroke prevention through timely anticoagulation remains crucial, though the ideal timing, especially for new-onset AF, needs further research. Additionally, ED discharge protocols and follow-up care for AF patients are often inconsistent, leaving many without proper long-term management. Integration of emerging therapies, including direct oral anticoagulants and advanced antiarrhythmic drugs, shows potential but remains uneven across EDs. Innovative multidisciplinary models, such as “AF Heart Teams” and observation units, could enhance care but face practical challenges in implementation. This review underscores the need for targeted research to refine AF management, optimize discharge protocols, and incorporate novel therapies effectively. Standardizing ED care for AF could significantly reduce stroke risk, lower readmission rates, and improve overall patient outcomes.

## 1. Introduction

Atrial fibrillation (AF), the most common sustained arrhythmia in clinical practice, is a major global driver of cardiovascular morbidity and mortality [1]. With an estimated 60 million people affected worldwide, the prevalence of AF is projected to increase as populations age and diagnostic capabilities improve [2]. AF is linked to a five-fold increase in stroke risk, a three-fold increase in heart failure, and a two-fold rise in mortality, imposing a substantial burden on healthcare systems, especially emergency departments (EDs) [3,4,5].

For many individuals with AF, the ED is the primary point of care, often due to acute symptom onset, such as palpitations, dizziness, or chest pain, or secondary complications, like thromboembolism or heart failure exacerbation [6,7]. ED clinicians play a critical role in the early management of AF, making decisions that influence immediate outcomes and long-term prognosis. However, AF management approaches in the ED vary widely, influenced by resource availability, access to cardiology consultation, and the clinical characteristics of the patient.

Historically, rate control has been the preferred initial strategy in stable AF patients, given its simplicity and safety profile, while rhythm control—achieved through cardioversion—has been reserved for patients with recent-onset AF, symptomatic instability, or anticipated long-term rhythm control [8]. Recent evidence, however, suggests that early rhythm control may benefit certain AF patients, sparking renewed interest in its use in the ED [9].

The introduction of direct oral anticoagulants (DOACs) has transformed stroke prevention in AF by providing a safer, more convenient alternative to vitamin K antagonists (VKAs) that eliminates the need for frequent monitoring [10]. Yet, the initiation of anticoagulation in the ED remains a debated area, requiring careful risk–benefit evaluation for stroke prevention versus bleeding risk. This decision is further complicated by follow-up challenges, as delayed outpatient access to cardiology care often leads to suboptimal anticoagulation management after ED discharge [11]. The 2024 European Society of Cardiology (ESC) guidelines for the management of AF emphasize the need for timely anticoagulation, particularly in high-risk patients, and advocate for stroke prevention strategies that begin in the ED setting [12].

Effective discharge planning and follow up are essential in AF management but are inconsistently applied in the ED. Protocols vary, with some patients discharged after brief observation and others admitted for monitoring or further treatment [13]. AF patients often lack comprehensive follow up, leading to missed opportunities for optimal long-term management and an increased risk of recurrent events [14]. Observation units within the ED have shown potential for reducing hospital admissions and ensuring adequate monitoring, though evidence on their long-term effectiveness remains limited [15].

Personalized medicine and multidisciplinary care models are increasingly shaping AF management [16]. Personalized approaches, tailored to each patient’s clinical profile, have shown promise in chronic disease management and are now being adapted to AF care [17]. In addition, multidisciplinary teams—comprising emergency physicians, cardiologists, and primary care providers—offer a coordinated approach to managing the acute and chronic aspects of AF, though logistical barriers to such models in the ED remain significant.

Significant knowledge gaps persist in managing AF, where optimal strategies for rate versus rhythm control, anticoagulation timing, and multidisciplinary care are not well-defined. This review explores these gaps by synthesizing the current literature on ED management strategies, discharge protocols, novel therapies, and care models specific to AF. Identifying and addressing these gaps is essential to standardize care, improve outcomes, and reduce complications for patients with AF in the ED.

## 2. Methodology

### 2.1. Timeline of Included Articles

To ensure a comprehensive and focused review, a structured methodology was established for selecting articles. Articles were published between January 2009 and October 2024. This period was chosen to capture the most recent and relevant advancements in the management of atrial fibrillation, including the development of novel anticoagulation, updated guidelines for rhythm and rate control, and evolving strategies in emergency department care.

### 2.2. Search Strategy

A systematic search was conducted using electronic databases, including PubMed, Embase, and Cochrane Library. The search strategy employed combinations of keywords and MeSH terms such as “atrial fibrillation”, “emergency department”, “rhythm control”, “rate control”, “electrical cardioversion”, and “anticoagulation”.

Boolean operators (e.g., AND, OR) were used to refine the search. Articles were filtered based on publication date, English language, and relevance to the study objectives.

### 2.3. Inclusion and Exclusion Criteria

Inclusion criteria include (1) studies that evaluated the management of atrial fibrillation in the emergency department, (2) articles discussing rate and rhythm control strategies, including cardioversion, and (3) publications addressing anticoagulation strategies in acute and long-term care.

Exclusion criteria include (1) studies not specific to atrial fibrillation, (2) articles focusing exclusively on non-acute settings, and (3) publications in languages other than English.

## 3. Knowledge Gaps in AF Management in the ED

The literature review revealed several significant knowledge gaps in the management of AF within the ED setting. These gaps were identified across key areas such as rate versus rhythm control strategies, anticoagulation initiation, discharge protocols, multidisciplinary care approaches, and the integration of novel therapies. Below, we present a detailed exploration of these identified knowledge gaps, drawing on recent studies and clinical guidelines to elucidate current practices and areas where further research is needed.

### 3.1. Rate vs. Rhythm Control: Ongoing Controversy

**Figure 1:**
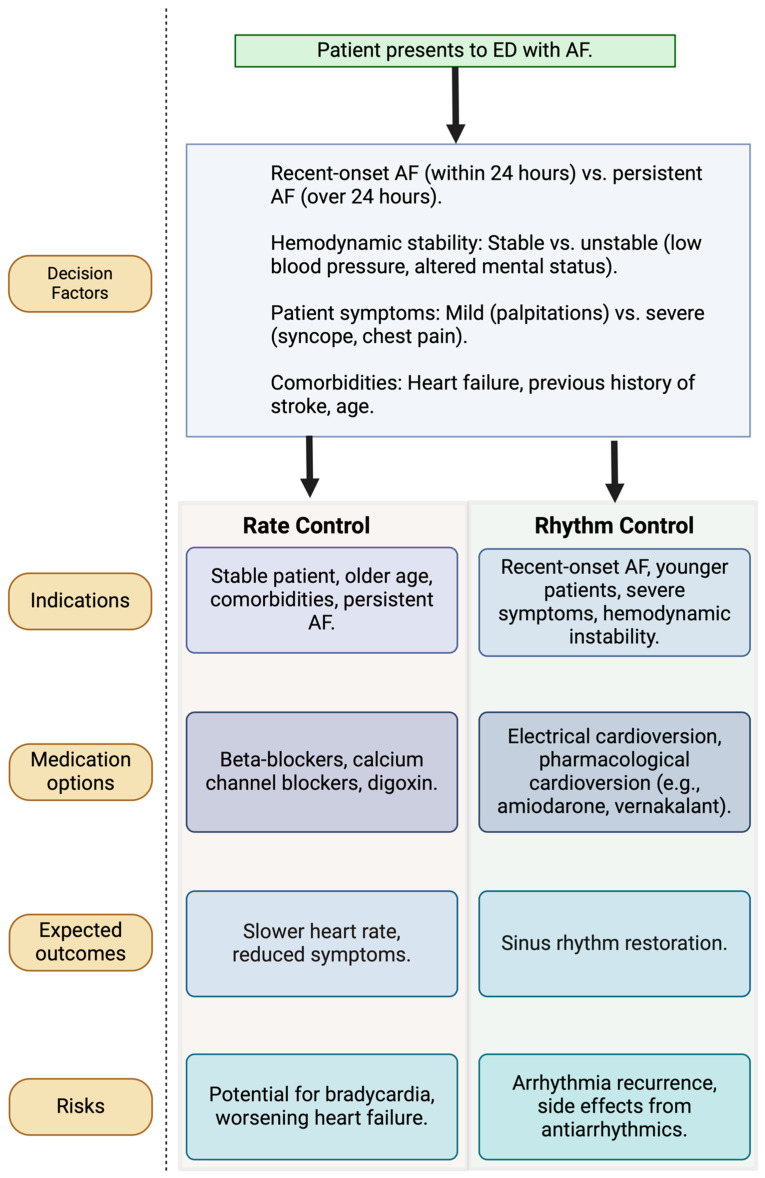
Rate versus rhythm control pathways. A flowchart illustrating the decision-making pathways for selecting rate or rhythm control in the emergency department. The diagram includes key triggers for each pathway, such as recent-onset atrial fibrillation and hemodynamic stability, along with potential outcomes and associated risks for each management strategy. AF, atrial fibrillation; ED, emergency department.

The debate over rate versus rhythm control remains one of the most contentious issues in AF management within the ED. While both approaches are outlined in clinical guidelines, real-world practice varies widely depending on patient presentation, local ED protocols, and the preferences of treating physicians. Atzema et al. (2015) highlighted that limited, conclusive data on whether rate or rhythm control offers superior benefits in the ED leads to inconsistent application of these strategies [18]. Some studies suggest that rate control may offer a safer initial option for stable AF patients, whereas others advocate for rhythm control as a means of potentially improving long-term outcomes, especially in younger, symptomatic patients (Figure 1) [12,19,20,21].

#### Impact of Anticoagulation Status on Rate vs. Rhythm Control Decisions

The choice between rate and rhythm control may also vary depending on a patient’s anticoagulation status. For patients already on anticoagulation, rhythm control strategies, such as cardioversion, can be pursued with a lower thromboembolic risk, especially in those with adequate anticoagulation coverage in the preceding weeks [12]. In contrast, anticoagulation-naïve patients present a more complex scenario. Rhythm control in these patients may necessitate initiation of anticoagulation and assessment of thromboembolic risk based on the duration of AF, with transesophageal echocardiography or delayed cardioversion considered in certain cases [12]. These nuances underscore the need for tailored approaches that integrate anticoagulation status into decision making (Table 1), further complicating the development of standardized protocols for ED settings.

### 3.2. Rate Control

Traditionally, rate control has been the preferred approach in the ED, particularly for stable AF patients, as it involves relatively straightforward and low-risk management with medications, such as beta-blockers and calcium channel blockers [12,18]. These drugs help to manage heart rate, a key factor in reducing symptoms and preventing complications associated with AF. However, consensus remains lacking on the optimal target heart rate for acute presentations in the ED. The AFFIRM trial recommended a target heart rate of <110 bpm for patients with persistent or permanent AF, as it was associated with satisfactory outcomes without increasing adverse events [22,23]. Yet, the ESC 2024 guidelines suggest stricter control, aiming for a resting heart rate of <80 bpm in patients with reduced left ventricular function, or those with cardiomyopathy, where tachycardia could exacerbate myocardial damage [12]. For post-surgical AF patients, a rate control target of <100 bpm has been suggested for asymptomatic individuals [24,25]. Despite these recommendations, the lack of evidence on optimal rate control targets in the ED setting underscores the need for further research, particularly to assess how heart rate control impacts long-term outcomes in this patient population.

### 3.3. Rhythm Control

Rhythm control, which can be achieved through electrical or pharmacological cardioversion, is increasingly considered for patients with recent-onset AF, significant symptoms, or those with high stroke risk [25,26,27]. Recent studies, including the EAST-AFNET 4 trial, show that early rhythm control reduced cardiovascular complications, like stroke and heart failure hospitalizations, lending support to its use in acute AF management [20]. Additionally, the RACE7 ACWAS trial indicated that many recent-onset AF cases might spontaneously resolve, suggesting a delayed approach to cardioversion may be feasible and beneficial [28]. According to the 2024 ESC guidelines, rhythm control is recommended early for symptomatic patients, especially those under 65 or with paroxysmal AF, as it may reduce recurrence and improve quality of life [12]. However, evidence specific to the ED setting regarding rhythm control’s impact on long-term AF recurrence, patient quality of life, and readmission rates remains sparse, and further studies are necessary to guide ED-based decision making.

#### Urgent Indications for Electrical Cardioversion

Electrical cardioversion (ECV) is considered urgent in specific scenarios where rapid restoration of sinus rhythm is critical. A common cause is hemodynamic instability, in which a patient presents acute AF associated with hypotension, heart failure, or other manifestations of compromised end-organ perfusion. It is also considered urgent in pre-excitation syndromes, such as AF, with Wolff–Parkinson–White syndrome, where irregular conduction through accessory pathways can precipitate ventricular fibrillation. It is often the next therapeutic consideration in patients with severe symptomatic AF refractory to pharmacologic interventions.

In the ED, ECG is generally feasible and highly effective, particularly for patients presenting within the “safe window” of <24 h of AF onset, where thromboembolic risk is relatively low, as suggested by the 2024 ESC guidelines [12]. However, feasibility may be influenced by several factors. Firstly, it is dependent on resource availability—ECV requires trained personnel, equipment (defibrillators), and availability of sedation/anesthesia support, which may vary across institutions. It is also dependent on local anticoagulation protocols. For patients with >24 h of AF duration or unknown timing, adequate anticoagulation or transesophageal echocardiography is likely required to rule out left atrial thrombus, which can delay the procedure. Another important consideration is patient tolerance and risks; as procedural sedation carries its own risks, particularly in older patients or those with significant comorbidities, and must be weighed against the benefits of rhythm control. In summary, while ECV is an effective and often urgent intervention in specific clinical situations, its application in the ED requires a multidisciplinary approach, adherence to safety protocols, and appropriate patient selection.

## 4. Anticoagulation and Stroke Prevention

Stroke prevention is a cornerstone of AF management, and timely initiation of anticoagulation is critical for patients at high risk of thromboembolism. However, the initiation of anticoagulation therapy in the ED remains inconsistent, particularly in patients with recent-onset AF or those without clear follow-up plans.

### 4.1. Timing of Anticoagulation

The decision to initiate anticoagulation in recent-onset AF cases (less than 48 h) is often contentious due to the balance required between stroke prevention and bleeding risks. For patients with a high CHA_2_DS_2_-VA score (Table 2), current guidelines favor early initiation of anticoagulation, even when cardioversion is planned [25]. The ESC 2024 guidelines reinforce this approach, especially for those presenting with AF under 24 h, where immediate anticoagulation may provide substantial benefits in reducing thromboembolic events [12]. Despite this, ED clinicians frequently hesitate to initiate anticoagulation in the absence of immediate follow up, as bleeding risks become difficult to monitor and manage outside a controlled setting [29]. Weant et al. (2020) found that while guidelines advocate for early anticoagulation, a considerable number of AF patients are discharged from the ED without stroke prevention therapy, highlighting gaps between guideline recommendations and real-world practice [30].

### 4.2. Choice of Anticoagulant

The emergence of DOACs has reshaped stroke prevention strategies in AF, with drugs such as apixaban, rivaroxaban, and dabigatran demonstrating superior stroke prevention and reduced bleeding risk compared to VKAs [31]. Landmark trials, such as ARISTOTLE and ROCKET-AF, have supported DOACs as first-line agents for non-valvular AF [32,33]. Despite these findings, research on DOAC initiation in the ED setting remains limited, particularly regarding patient selection criteria, as well as strategies for post-discharge anticoagulation management. This lack of clear guidelines on DOAC use in the ED has contributed to varied practices and points to a need for targeted research to clarify DOAC initiation protocols for ED patients with AF.

### 4.3. Risk Stratification and HAS-BLED Score

The HAS-BLED score is an important tool for assessing bleeding risk in patients with AF who are being considered for anticoagulation therapy (Table 3) [34,35]. A score of ≥3 indicates a high bleeding risk, necessitating close monitoring and careful consideration of modifiable risk factors such as hypertension, labile INR, and concomitant use of medications that increase bleeding risk. While the HAS-BLED score does not preclude anticoagulation, it guides clinicians in optimizing therapy and weighing the risks versus benefits.

### 4.4. Special Populations in Pharmacotherapy Selection

Pharmacotherapy selection for AF often requires addressing the unique needs of special populations such as those with chronic kidney disease, frailty syndrome, and cancer (Table 4). These conditions compound the complexities of anticoagulation management, emphasizing the need for a tailored approach informed by clinical tools, such as the HAS-BLED score, and comprehensive patient assessments. Proactive management of modifiable risk factors and collaboration across specialties are critical in ensuring optimal outcomes in these vulnerable groups.

#### 4.4.1. Chronic Kidney Disease

Patients with chronic kidney disease present unique challenges in the use of DOACs. Renal function must be assessed before initiating therapy, as many of these anticoagulants are partially excreted by the kidneys [36]. Dose adjustments based on the estimated glomerular filtration rate are critical, with some DOACs contraindicated in severe chronic kidney disease or end-stage renal disease [36,37,38]. In these cases, warfarin remains a commonly used alternative despite its narrower therapeutic window.

#### 4.4.2. Frailty Syndrome

Older patients with frailty syndrome are at increased risk of both thromboembolic and bleeding events. Pharmacotherapy selection in this population requires an individualized approach, balancing stroke prevention with bleeding risk [39]. Lower DOAC regimens or careful titration of warfarin may be appropriate in select cases, supported by frequent monitoring and geriatric assessment to minimize adverse outcomes.

#### 4.4.3. Cancer-Associated AF

Cancer patients with AF face a complex interplay of thromboembolic risk from malignancy and treatment-associated bleeding risks. DOACs, particularly those with a lower propensity for gastrointestinal bleeding, have shown promise in cancer-associated thrombosis and AF [40]. However, in patients with high bleeding risk or those undergoing active chemotherapy, low-molecular-weight heparins may be preferred [41]. A multidisciplinary approach, incorporating oncology and hematology expertise, is essential in these cases.

## 5. Discharge Protocols and Follow-Up Care

**Figure 2:**
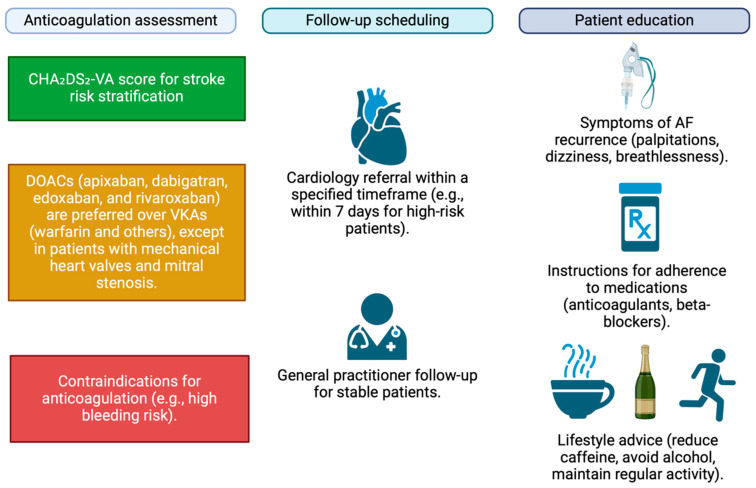
Standardized discharge protocol for patients with atrial fibrillation. Key steps include evaluating anticoagulation needs, arranging follow-up appointments, and providing patient education on recognizing signs of AF recurrence.

One of the most critical challenges in managing AF in the ED is determining appropriate discharge protocols and ensuring adequate follow-up care. There are existing guidelines outlining a standardized discharge protocol for AF patients (Figure 2). A study in Canada identified that follow up within 7 days versus follow up between days 8 and 30 after ED discharge was associated with a significant reduction in all-cause mortality by 1 year and a lower risk of cardiovascular hospitalizations within 1 year [42]. However, it is important to acknowledge that this recommendation may not be universally feasible or appropriate across all healthcare settings. There is a wide variation in how and when AF patients are discharged from the ED, leading to potential gaps in care.

### 5.1. Observation Units

Many EDs have introduced observation units to extend the monitoring of AF patients without necessitating full hospital admission [15,43]. This approach has shown promise in reducing unnecessary admissions while still providing a safety net for patients at risk. Bellew et al. (2016) reported that observation units can reduce admissions and improve short-term outcomes for AF patients, though evidence of their long-term impact on stroke prevention or AF recurrence is limited [15]. Further research is needed to clarify the role of observation units in managing AF patients and determine how they impact outcomes, like readmission rates and recurrence, over extended periods.

### 5.2. Post-Discharge Follow up

One of the major challenges for ED-based AF management is ensuring that discharged patients receive adequate follow-up care, particularly when outpatient access to cardiology services is limited. The ORBIT-AF registry highlighted that nearly 40% of AF patients discharged from the ED do not receive adequate anticoagulation, increasing their stroke risk significantly [44]. The 2024 ESC guidelines advocate for structured follow-up pathways to ensure that patients discharged with AF receive prompt follow up with cardiologists or primary care providers [12]. Unfortunately, barriers such as limited healthcare resources and gaps in outpatient continuity often result in suboptimal follow up, raising the risk of recurrence, inadequate anticoagulation management, and higher readmission rates.

## 6. Multidisciplinary Care Models

The management of AF in the ED increasingly requires a collaborative approach, involving not only emergency physicians but also cardiologists, electrophysiologists, and primary care providers. Multidisciplinary care models, such as the “AF Heart Team” concept, have shown promise in improving patient outcomes, but their implementation remains inconsistent [45].

### 6.1. Integrated Care Pathways

The 2024 ESC guidelines emphasize the importance of integrated care pathways that streamline the transition from ED to outpatient management, facilitating anticoagulation management, rhythm control decisions, and longer-term monitoring [12]. However, practical implementation of such pathways remains challenging in real-world settings, particularly in resource-limited areas. Financial, logistical, and personnel constraints limit the establishment of integrated care models in many EDs, highlighting an area where health policy could play a significant role in improving AF care.

### 6.2. Multidisciplinary Teams

The RACE II trial demonstrated that a coordinated, multidisciplinary rate control approach achieved outcomes comparable to those of rhythm control strategies [46]. A study by Ptaszek et al. (2016) showed that a multidisciplinary AF management model could reduce hospital admissions and shorten ED stays, though these models are underutilized in many healthcare systems due to logistical and financial barriers [47]. Expanding these collaborative models in ED settings could enhance AF patient outcomes, yet further research is needed to develop scalable, cost-effective multidisciplinary care approaches.

## 7. Integration of Novel Therapies

The introduction of novel therapies, including DOACs and newer antiarrhythmics, like vernakalant and dronedarone, has significantly advanced AF management. These innovations address key challenges of traditional treatments, improving safety, convenience, and effectiveness. However, consistent integration of these therapies into ED protocols remains uneven, with studies like Vinson et al. (2012) noting gaps in their real-world implementation, which limits the full benefits of these medications in acute settings [48]. This section explores the current application, benefits, and challenges of DOACs and antiarrhythmic drugs in the ED management of AF and highlights the need for ongoing research to optimize their use [48].

### 7.1. DOACs

DOACs have transformed stroke prevention in AF, offering a safer and more practical alternative to traditional VKAs due to their reduced bleeding risk, fewer drug interactions, and no requirement for regular monitoring [49]. The ESC 2024 guidelines now recommend DOAC initiation as early as the ED phase for eligible AF patients, particularly those at high stroke risk [12]. Despite this, many EDs hesitate to start DOAC therapy, primarily due to concerns about continuity of care and anticoagulation monitoring after discharge, especially for patients with inconsistent access to outpatient follow up [12]. Additionally, the lack of universally standardized protocols for DOAC initiation in the ED setting contributes to variability in practice.

Emerging evidence underscores the safety and efficacy of DOAC initiation in the ED for eligible patients, with studies showing reduced stroke risk without significantly increasing bleeding complications when DOACs are started early in high-risk patients [50]. However, further research is needed to evaluate the long-term outcomes of ED-initiated DOAC therapy, particularly in settings where follow-up care is limited or uncertain. Investigating the impact of structured follow-up protocols on DOAC safety and patient adherence may help inform future guidelines and streamline DOAC use in the ED.

### 7.2. Antiarrhythmic Drugs

Newer antiarrhythmic drugs, such as vernakalant and dronedarone, provide additional options for rhythm control, especially in patients with recent-onset AF [51]. These drugs offer potential advantages over traditional antiarrhythmics by promoting faster cardioversion with a lower incidence of adverse effects. Vernakalant, for instance, has demonstrated higher efficacy in achieving sinus rhythm compared to ibutilide, as shown in studies like that by Simon et al. [52]. This rapid, actionable safety profile makes vernakalant a promising choice for ED-based cardioversion, particularly in patients who require urgent rhythm control [53].

Despite these benefits, the use of novel antiarrhythmics in the ED remains limited. Concerns about real-world efficacy, cost, and the potential for side effects in high-risk populations contribute to reluctance in their routine adoption. Furthermore, while ESC guidelines support the selective use of these agents, variability in clinician familiarity and institutional protocols has led to inconsistent application. Additional large-scale studies evaluating the effectiveness and safety of these drugs in ED settings are needed, as well as clinical trials focusing on specific patient subgroups, such as those with comorbidities like heart failure or structural heart disease.

## 8. Risk Factor Modification

Management of AF extends beyond pharmacological and procedural interventions to include comprehensive risk factor modification, which has emerged as a cornerstone of AF care. Evidence from landmark studies highlights the profound impact of addressing modifiable risk factors such as obesity, obstructive sleep apnea (OSA), hypertension, diabetes, and lifestyle factors, like physical inactivity and alcohol consumption.

### 8.1. Weight Management

Obesity is a major risk factor in AF, contributing to structural and electrical remodeling of the atria. The LEGACY trial demonstrated that sustained weight loss of >10% significantly reduced AF burden, with many patients achieving long-term rhythm control without invasive therapy [54]. Similarly, the CARDIO-FIT study emphasized the synergistic benefits of weight loss and improved cardiorespiratory fitness, showing a marked reduction in AF episodes among participants who achieved both targets [55]. These findings underscore the importance of integrating structured weight management programs into AF treatment pathways, particularly for obese patients.

Practical implementation of weight management involves lifestyle interventions, such as dietary modifications, structured exercise programs, and, in some cases, bariatric surgery, particularly for patients with severe obesity or obesity-related comorbidities. Incorporating these strategies into clinical practice requires a multidisciplinary approach involving cardiologists, primary care providers, and nutrition and fitness experts.

### 8.2. OSA Management

OSA is highly prevalent among AF patients, with studies estimating its presence in up to 50% of individuals with AF. The Sleep Heart Health Study and subsequent trials have established OSA as an independent risk factor for AF development, progression, and recurrence post-treatment [56,57]. Continuous positive airway pressure therapy has demonstrated efficacy in reducing AF recurrence rates, particularly in patients undergoing rhythm control strategies like catheter ablation [58].

In clinical practice, screening for OSA using tools, such as the STOP-BANG questionnaire, should be integrated into routine AF assessments, particularly for high-risk individuals [59,60,61,62]. Early diagnosis and adherence to CPAP therapy are critical for optimizing outcomes and preventing recurrent AF episodes [63].

### 8.3. Comprehensive Lifestyle Modification

Beyond weight and OSA, other lifestyle factors significantly impact AF outcomes. High blood pressure control, glycemic optimization in diabetes, alcohol reduction, and smoking cessation have all been shown to reduce AF burden and improve overall cardiovascular health [64]. The ARREST-AF study, for instance, demonstrated that aggressive risk factor modification, including hypertension and glycemic control, resulted in improved rhythm control outcomes post-ablation [65].

## 9. Global Disparities in AF Management

The management of AF varies significantly worldwide, especially between high-income countries (HICs) and low- and middle-income countries (LMICs). These differences reflect disparities in healthcare infrastructure, resources, and adherence to evidence-based guidelines, which affect patient access to timely diagnosis, effective treatment, and follow-up care. Addressing these gaps is essential to improving AF outcomes on a global scale.

### 9.1. Access to Care

Access to healthcare resources for AF management varies widely, with LMICs facing substantial limitations compared to HICs. In LMICs, patients often encounter barriers such as fewer specialized healthcare providers, limited availability of advanced diagnostic tools, and restricted access to treatments like DOACs and antiarrhythmic drugs [66]. For instance, in high-income countries, early use of DOACs is now common practice for stroke prevention in AF, but in LMICs, access to these medications is frequently restricted due to high costs and limited availability. Additionally, regional disparities within countries—especially between urban and rural areas—compound these challenges, with rural patients often having even less access to specialist care and advanced therapies.

Studies, such as Oldgren et al. (2014), reveal significant global variation in AF treatment and outcomes, underscoring a particular need for standardized protocols adaptable to local resource constraints [67]. In many LMICs, adherence to clinical guidelines is hindered not by a lack of awareness but by a lack of infrastructure and resources, making it difficult for clinicians to implement guideline-based care. Strategies such as task shifting, where non-specialist healthcare providers receive training in AF management, may help expand access in regions where specialists are scarce [68]. Additionally, international partnerships and funding initiatives can support increased access to essential medications and diagnostic tools, helping to close the gap in AF care [69].

### 9.2. Telemedicine and Remote Monitoring

Telemedicine and remote monitoring have emerged as promising tools to improve AF management, especially in resource-limited settings. The 2024 ESC guidelines advocate for telemedicine to enhance continuity of care and reduce barriers to follow up, particularly in underserved areas [12]. Through telemedicine, patients can access virtual consultations with specialists, receive timely medication adjustments, and have symptoms monitored remotely, which is crucial for the early detection of AF recurrence and the prevention of complications [70].

Remote monitoring devices, such as wearable ECG patches and smartphone apps, allow continuous tracking of heart rhythm and can alert both patients and providers to irregularities, facilitating early intervention [71]. However, challenges remain in ensuring equitable access to these technologies, as costs and infrastructure limitations still prevent widespread implementation in many LMICs. Furthermore, there is a need for more research on the effectiveness of telemedicine and remote monitoring in improving AF outcomes across diverse populations, particularly to understand how these technologies can be adapted to local needs and integrated into existing healthcare systems in LMICs.

Pilot programs that combine telemedicine with community health worker support have shown promise in LMICs, where remote monitoring is combined with in-person follow up and patient education provided by local healthcare workers [72]. This hybrid approach could help overcome technology barriers and optimize AF management in settings where full telemedicine integration remains challenging.

### 9.3. Standardizing Global AF Guidelines and Care Protocols

While international guidelines provide an evidence-based foundation for AF management, significant differences in healthcare systems and resources necessitate adaptable, region-specific protocols. The development of simplified guidelines tailored to resource-limited environments can help standardize AF care globally while ensuring that guidelines are practical for LMIC settings. In addition, international initiatives, such as the World Health Organization’s Global Hearts Initiative, aim to strengthen the management of cardiovascular diseases, like AF, by building local capacity, training healthcare providers, and improving access to essential medicines.

Expanding such initiatives could help to reduce disparities and establish a foundation for universal AF care standards that are both effective and feasible worldwide. Partnerships among international health organizations, governments, and non-governmental organizations are essential for sustaining these efforts, providing financial support, and ensuring equitable access to treatment.

### 9.4. Addressing Socioeconomic Barriers and Health Literacy

Socioeconomic barriers, including poverty and limited health literacy, further exacerbate global disparities in AF management. Patients with lower socioeconomic status often experience delayed diagnosis, poorer access to medications, and lower rates of follow-up care, even in HICs [73]. Enhancing health literacy through patient education programs can empower individuals to recognize AF symptoms, understand the importance of treatment adherence, and seek care early, which is critical to preventing complications, such as stroke and heart failure.

Community-based programs focused on education, such as workshops and public awareness campaigns, can help bridge these gaps, especially in LMICs [74]. In addition, culturally sensitive approaches that involve family members in the care process may improve patient engagement and adherence to treatment plans, especially in communities with low health literacy [75].

## 10. Discussion

The findings from this review highlight several critical knowledge gaps in the management of AF in the ED. Despite advancements in clinical guidelines and therapeutic options, there remains significant variability in how AF is treated in the acute care setting, with key areas of uncertainty affecting both immediate and long-term patient outcomes. Addressing these gaps is essential for optimizing AF care and ensuring that patients receive timely, evidence-based interventions that reduce the risk of complications such as stroke, heart failure, and mortality.

### 10.1. Rate vs. Rhythm Control: Tailoring Treatment to Patient Profiles

The ongoing debate between rate and rhythm control in the ED is reflective of broader uncertainties in AF management. Rate control, traditionally favored for its simplicity and lower immediate risk, remains the predominant strategy in many EDs, particularly for stable patients. However, the introduction of more refined criteria in the 2024 ESC guidelines, advocating for earlier rhythm control in select patient groups (e.g., younger patients or those with symptomatic, recent-onset AF), suggests a shift toward more aggressive management in certain scenarios.

The lack of consensus on which patients benefit most from rhythm control versus rate control, particularly in the ED where decision making must often be swift, underscores the need for more robust clinical data. While rhythm control may improve long-term outcomes, such as quality of life and AF recurrence, in certain patients, particularly those with early-stage or paroxysmal AF, its broader application in the ED is still debated due to the risk of complications and the resources required for cardioversion.

Future research should focus on randomized controlled trials that compare rate and rhythm control strategies specifically in the ED, with a focus on different AF subtypes (e.g., paroxysmal vs. persistent) and patient demographics (e.g., age, comorbidities). These studies should assess not only acute outcomes, such as symptom resolution and hemodynamic stability, but also long-term endpoints, such as recurrence, hospitalization rates, and patient-centered outcomes, like quality of life.

This review also highlights the nuanced decision making required in anticoagulation therapy for patients with AF, particularly in those with high bleeding risk, renal impairment, frailty, or cancer. Incorporating tools like the HAS-BLED score and tailoring therapy based on individual patient factors ensures that stroke prevention strategies are both safe and effective. Future research should focus on refining risk stratification models and developing anticoagulation options with broader safety profiles for these high-risk populations.

### 10.2. Anticoagulation in the ED: Striking a Balance Between Efficacy and Safety

The decision to initiate anticoagulation in the ED, particularly for patients with recent-onset AF, remains a critical area of uncertainty. While the 2024 ESC guidelines advocate for early anticoagulation in patients at high risk of stroke (e.g., CHA_2_DS_2_-VA score ≥ 2) (Table 2), in practice, ED physicians may be hesitant to initiate therapy due to concerns about bleeding, lack of outpatient follow up, or uncertainty regarding the need for cardioversion.

DOACs have simplified stroke prevention in AF, offering advantages over VKAs, such as fewer drug interactions and no requirement for routine monitoring. However, the integration of DOACs into ED management protocols is still inconsistent, particularly in settings where follow-up care may be uncertain. This hesitation can result in suboptimal stroke prevention, with patients discharged without adequate anticoagulation therapy, leading to increased stroke risk.

Further research is needed to explore the safety and efficacy of initiating DOACs in the ED, particularly for patients with recent-onset AF. Studies should assess the impact of early anticoagulation on long-term outcomes, such as stroke prevention, as well as the risks associated with bleeding in patients without immediate access to follow-up care. Additionally, the development of ED-specific protocols for anticoagulation initiation, tailored to different patient profiles and risk factors, could help standardize care and reduce variability in practice.

### 10.3. Discharge Protocols and Post-Discharge Monitoring: Addressing the Transition of Care

One of the most significant gaps in AF management is the lack of standardized discharge protocols and structured follow-up care after an ED visit. Current discharge practices vary widely, with some patients being admitted for further monitoring, while others are discharged home with limited or no follow up. This inconsistency can result in patients either being over-treated with unnecessary admissions or under-treated with inadequate monitoring, increasing the risk of stroke, AF recurrence, or other complications.

The use of observation units, as suggested by several studies, has shown the potential to reduce unnecessary hospital admissions while ensuring that patients are adequately monitored before discharge [76]. However, the effectiveness of these units in improving long-term outcomes, such as recurrence rates, adherence to anticoagulation therapy, and patient satisfaction, has not been thoroughly evaluated.

Additionally, many AF patients discharged from the ED do not receive adequate follow-up care. This is particularly concerning for patients started on anticoagulation therapy, as they require careful monitoring for bleeding risks and stroke prevention. The 2024 ESC guidelines emphasize the importance of integrating structured care pathways that include timely follow up with cardiologists or primary care physicians, yet real-world implementation of these pathways remains limited.

Future research needs to develop and validate standardized discharge protocols that balance safety with efficiency. These protocols should include clear criteria for when patients can be safely discharged versus admitted for further observation, along with detailed plans for post-discharge monitoring. Additionally, the role of telemedicine and remote monitoring tools in providing follow-up care for AF patients discharged from the ED should be explored, particularly in rural or underserved areas where access to specialty care may be limited.

### 10.4. Multidisciplinary Care Models: A Collaborative Approach to AF Management

**Figure 3:**
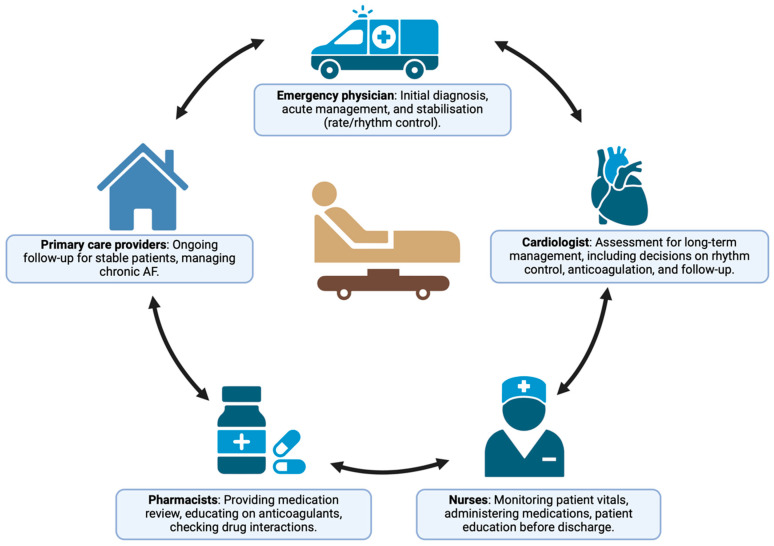
Multidisciplinary care model for atrial fibrillation management in the emergency department. The roles of various healthcare professionals—emergency physicians, cardiologists, nurses, pharmacists, and primary care providers—in the coordinated management of atrial fibrillation in the emergency department. This model emphasizes how teamwork across disciplines can reduce hospital admissions and enhance patient outcomes.

The integration of multidisciplinary care models, such as the “AF Heart Team”, offers a promising approach to improving AF management in the ED. These teams typically include emergency physicians, cardiologists, electrophysiologists, and other healthcare providers who work together to develop personalized care plans for AF patients. The 2024 ESC guidelines emphasize the importance of these collaborative models, particularly for high-risk patients who may benefit from early rhythm control or advanced therapeutic options (Figure 3) [12].

Despite the potential benefits, the implementation of multidisciplinary care models in the ED is still limited by logistical challenges, such as the availability of specialists and the financial resources required to sustain these teams. Additionally, there is a lack of data on the cost effectiveness of multidisciplinary care in the ED, particularly in terms of reducing hospital admissions and improving long-term outcomes.

Further research is needed to assess the feasibility and scalability of multidisciplinary care models in different healthcare settings, particularly in low-resource environments. Studies should evaluate the impact of these models on patient outcomes, such as hospital readmission rates, the recurrence of AF, and overall quality of care. Additionally, the financial implications of implementing multidisciplinary teams should be explored, with a focus on cost effectiveness and resource allocation.

### 10.5. Integration of Novel Therapies: Opportunities and Challenges

The introduction of novel therapies, such as DOACs and new antiarrhythmic drugs, has transformed AF management, offering safer and more effective options for stroke prevention and rhythm control. However, their integration into ED practice remains inconsistent, particularly in settings where access to follow-up care may be limited.

DOACs have become the preferred option for stroke prevention in non-valvular AF due to their ease of use and improved safety profile compared to VKAs. However, many EDs have yet to fully integrate DOACs into their protocols, particularly for patients with recent-onset AF or those who require cardioversion. Similarly, newer antiarrhythmic drugs, such as vernakalant and dronedarone, offer faster cardioversion rates with fewer side effects, but their use in the ED remains limited due to a lack of familiarity and experience among emergency physicians.

More research is needed to evaluate the use of novel therapies in the ED setting, particularly regarding their safety, efficacy, and cost effectiveness. Studies should focus on developing guidelines for the initiation of DOACs and newer antiarrhythmics in the ED, with consideration for patient-specific factors such as stroke risk, bleeding risk, and the likelihood of AF recurrence. Additionally, educational initiatives for emergency physicians on the use of these novel therapies could help facilitate their integration into routine practice.

### 10.6. Addressing Global Disparities in AF Management

Regional and global disparities in AF management present significant challenges to providing consistent, high-quality care for AF patients in the ED. In LMICs, access to advanced therapies, such as DOACs and catheter ablation, is often limited, leading to poorer outcomes for AF patients. Additionally, the lack of structured follow-up care and limited availability of cardiology services further exacerbate these disparities.

The 2024 ESC guidelines call for greater efforts to reduce these disparities by standardizing care protocols and improving access to affordable therapies. Telemedicine and remote monitoring technologies offer potential solutions for bridging the gap in follow-up care, particularly in rural and underserved areas. However, the effectiveness of these technologies in improving long-term outcomes for AF patients remains unclear.

In smaller or resource-constrained healthcare centers, the availability of medication recommended as first-line therapy for AF in guidelines may be limited. This often necessitates the use of alternative agents that are not explicitly prioritized in the guidelines, creating potential challenges in maintaining evidence-based care. Clinicians in such settings must adapt their treatment strategies by weighing the benefits of available medications against their potential risks and effectiveness. For instance, in the absence of newer anticoagulants, older agents, such as warfarin, may still play a pivotal role despite their less favorable risk profile.

To mitigate these challenges, continuous medical education and tailored training programs for healthcare providers in smaller centers are essential. These programs can focus on optimizing the use of available medications, integrating local resource constraints into care strategies, and fostering innovation in treatment delivery.

Comparative studies needed to be conducted by clinical researchers to assess how regional differences in AF management impact patient outcomes, including stroke rates, hospital admissions, and long-term quality of life. Additionally, research should focus on evaluating the effectiveness of telemedicine and remote monitoring solutions in providing follow-up care for AF patients discharged from the ED, particularly in low-resource settings.

There is also a need for guideline committees to consider the variability in resource availability when formulating recommendations. Providing alternative therapeutic options or tiered guidance can better support clinicians working in diverse healthcare environments.

## 11. Conclusions

The management of AF in the ED remains a complex and multifaceted challenge, with significant gaps in the current understanding of optimal treatment strategies. Addressing these knowledge gaps will require targeted research efforts that focus on the comparative effectiveness of rate versus rhythm control, the timing and safety of anticoagulation initiation, the development of standardized discharge protocols, and the integration of novel therapies and multidisciplinary care models.

The 2024 ESC guidelines provide important updates that can guide future research and clinical practice, but there is still work to be performed to optimize AF management in the ED. By addressing these gaps, healthcare providers can improve both short- and long-term outcomes for AF patients, reduce the risk of complications, and standardize care practices across diverse healthcare settings.

## Figures and Tables

**Table 1:** The interplay between anticoagulation status and the immediate management strategy in the ED. Tailored approaches are based on individual patient profiles and risk. AF, atrial fibrillation; TEE, transesophageal echocardiogram.

Aspect	Patients on Anticoagulation	Anticoagulation-Naïve Patients
Stroke Risk Mitigation	Stroke risk already reduced by existing therapy	Stroke risk must be actively addressed
Rhythm Control	Safer and preferred for symptom relief if indicated	Dependent on AF duration; requires anticoagulation or TEE
Rate Control	Often adequate for stable patients	Conservative approach preferred if AF > 24 h
Anticoagulation Approach	No new initiation required	Initiation required, often in the ED

**Table 2:** Updated definitions for the CHA_2_DS_2_-Va score. CAD, coronary artery disease; CHA_2_DS_2_-Va chronic heart failure, hypertension, age ≥ 75 years (2 points), diabetes mellitus, prior stroke/transient ischaemic attack/arterial thromboembolism (2 points), vascular disease, age 65–74 years; HFmrEF, heart failure with mildly reduced ejection fraction; HFpEF, heart failure with preserved ejection fraction; HFrEF, heart failure with reduced ejection fraction; LVEF, left ventricular ejection fraction; PVD, peripheral vascular disease.

CHA_2_DS_2_-Va Component	Description
C: Chronic heart failure	Presence of heart failure symptoms and signs, regardless of left ventricular ejection fraction (includes HFpEF, HFmrEF, and HFrEF), or an asymptomatic LVEF of ≤40%.
H: Hypertension	Presence of heart failure symptoms and signs, regardless of left ventricular ejection fraction (includes HFpEF, HFmrEF, and HFrEF), or an asymptomatic LVEF of ≤40%.
A: Age 75 years or above	Age is a strong independent risk factor for ischemic stroke. Although stroke risk increases continuously with age, patients 75 or older are given 2 points to account for higher risk.
D: Diabetes mellitus	Diagnosis of either type 1 or type 2 diabetes based on established criteria or treatment with glucose-lowering medications.
S: Prior stroke, TIA, or arterial thromboembolism	A history of these conditions significantly raises recurrence risk, warranting a weighting of 2 points.
V: Vascular disease	This includes the following: - Coronary artery disease (CAD). History of myocardial infarction, angina, coronary revascularization (surgical or percutaneous), or significant CAD findings on imaging or angiography. - Peripheral vascular disease (PVD). Includes intermittent claudication, past revascularization for PVD, interventions on the abdominal aorta, or imaging evidence of complex aortic plaque (features like mobility, ulceration, pedunculation, or thickness ≥4 mm).
A: Age 65–74 years	An additional point is assigned for individuals aged 65 to 74.

**Table 3:** HASBLED score. A score of ≥3 indicates a high bleeding risk. ALT, alanine aminotransferase; AP, alkaline phosphatase; AST, aspartate transferase; INR, international normalized ratio; NSAID, non-steroidal anti-inflammatory drug.

HAS-BLED Component	Description
Hypertension	Uncontrolled, >160 mmHg systolic
Abnormal renal/liver function (1 point each).	Renal disease: Dialysis, transplant, creatinine > 2.26 mg/dL or >200 µmol/L. Liver disease: Cirrhosis or bilirubin > 2x normal with AST/ALT/AP > 3x normal
Stroke	History of stroke
Bleeding	Prior major bleeding or predisposition to bleeding
Labine INR	Unstable/high INRs, time in therapeutic range < 60%
Elderly	Age > 65 years old, extreme frailty
Drugs or alcohol (1 point each)	Drugs: concomitant antiplatelet or NSAID use. Alcohol use > 7 drinks/week

**Table 4:** Practical considerations for anticoagulant use in special populations. AF = atrial fibrillation, DOAC = direct oral anticoagulant.

Population	Key Considerations	Preferred Approach
Chronic kidney disease	Renal function monitoring; dose adjustment for DOACs; warfarin for severe chronic kidney disease	Adjusted dose DOACs or warfarin
Frailty syndrome	High risk of falls and bleeding; frequent INR monitoring with warfarin	Reduced dose DOACs or adjusted warfarin doses
Cancer-associated AF	Increased thromboembolic risk; bleeding risk with chemotherapy; gastrointestinal bleeding risk with some DOACs	DOACs with lower bleeding risk or low molecular weight heparin

## Data Availability

No new data were created.

## Abbreviations

AF	Atrial fibrillation
BP	Blood pressure
CAD	Coronary artery disease
CHA_2_DS_2_-VA	Chronic heart failure, hypertension, age ≥ 75 years (2 points), diabetes mellitus, prior stroke/transient ischaemic attack/arterial thromboembolism (2 points), vascular disease, age 65–74 years
DOAC	Direct oral anticoagulant
ECV	Electrical cardioversion
ED	Emergency department
ESC	European Society of Cardiology
HAS-BLED	Hypertension (uncontrolled, >160 mmHg systolic), renal disease (dialysis, transplant, creatinine > 2.26 mg/dL or >200 µmol/L), liver disease (cirrhosis or bilirubin > 2x normal with AST/ALT/AP > 3x normal), stroke history, prior major bleeding or predisposition to bleeding, labine INR (unstable/high INRs, time in therapeutic range < 60%), age > 65, medication usage predisposing to bleeding (aspirin, clopidogrel, NSAIDs, alcohol use > 7 drinks/week).
HFmrEF	Heart failure with mildly reduced ejection fraction
HIC	High-income country
HFpEF	Heart failure with preserved ejection fraction
HFrEF	Heart failure with reduced ejection fraction
LMIC	Low- and middle-income country
LVEF	Left ventricular ejection fraction
PVD	Peripheral vascular disease
VKA	Vitamin K antagonist
